# Ultrafast proton-coupled isomerization in the phototransformation of phytochrome

**DOI:** 10.1038/s41557-022-00944-x

**Published:** 2022-05-16

**Authors:** Yang Yang, Till Stensitzki, Luisa Sauthof, Andrea Schmidt, Patrick Piwowarski, Francisco Velazquez Escobar, Norbert Michael, Anh Duc Nguyen, Michal Szczepek, Florian Nikolas Brünig, Roland Rüdiger Netz, Maria Andrea Mroginski, Suliman Adam, Franz Bartl, Igor Schapiro, Peter Hildebrandt, Patrick Scheerer, Karsten Heyne

**Affiliations:** 1 Department of Physics, Freie Universität Berlin, Berlin, Germany; 2 Charité – Universitätsmedizin Berlin, Corporate Member of Freie Universität Berlin and Humboldt-Universität zu Berlin, Institute of Medical Physics and Biophysics, Group Protein X-ray Crystallography and Signal Transduction, Berlin, Germany; 3 Institut für Biologie, Biophysikalische Chemie, Humboldt-Universität zu Berlin, Berlin, Germany; 4 Institut für Chemie, Technische Universität Berlin, Berlin, Germany; 5 Fritz Haber Center for Molecular Dynamics Research, Institute of Chemistry, The Hebrew University of Jerusalem, Jerusalem, Israel

**Keywords:** Biochemistry, Biophysics, Chemical biology, Chemistry

## Abstract

The biological function of phytochromes is triggered by an ultrafast photoisomerization of the tetrapyrrole chromophore biliverdin between two rings denoted *C* and *D*. The mechanism by which this process induces extended structural changes of the protein is unclear. Here we report ultrafast proton-coupled photoisomerization upon excitation of the parent state (Pfr) of bacteriophytochrome Agp2. Transient deprotonation of the chromophore’s pyrrole ring *D* or ring *C* into a hydrogen-bonded water cluster, revealed by a broad continuum infrared band, is triggered by electronic excitation, coherent oscillations and the sudden electric-field change in the excited state. Subsequently, a dominant fraction of the excited population relaxes back to the Pfr state, while ~35% follows the forward reaction to the photoproduct. A combination of quantum mechanics/molecular mechanics calculations and ultrafast visible and infrared spectroscopies demonstrates how proton-coupled dynamics in the excited state of Pfr leads to a restructured hydrogen-bond environment of early Lumi-F, which is interpreted as a trigger for downstream protein structural changes.

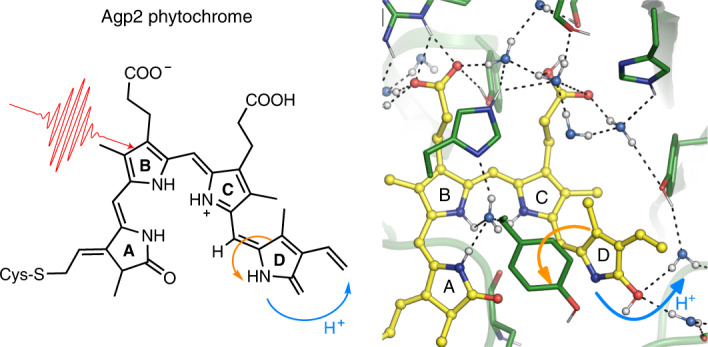

## Abstract (web summary)

Phytochromes regulate plant growth by sensing far-red light through the photoisomerization of their protein-bound chromophores. In the phytochrome Agp2, it has now been demonstrated that ultrafast proton-transfer occurs from the chromophore to a protein–water network before photoisomerization, inducing protein changes on the ultrafast timescale. These protein changes develop further on longer timescales, resulting in an activated protein conformation.

## Main

**Fig. 1:**
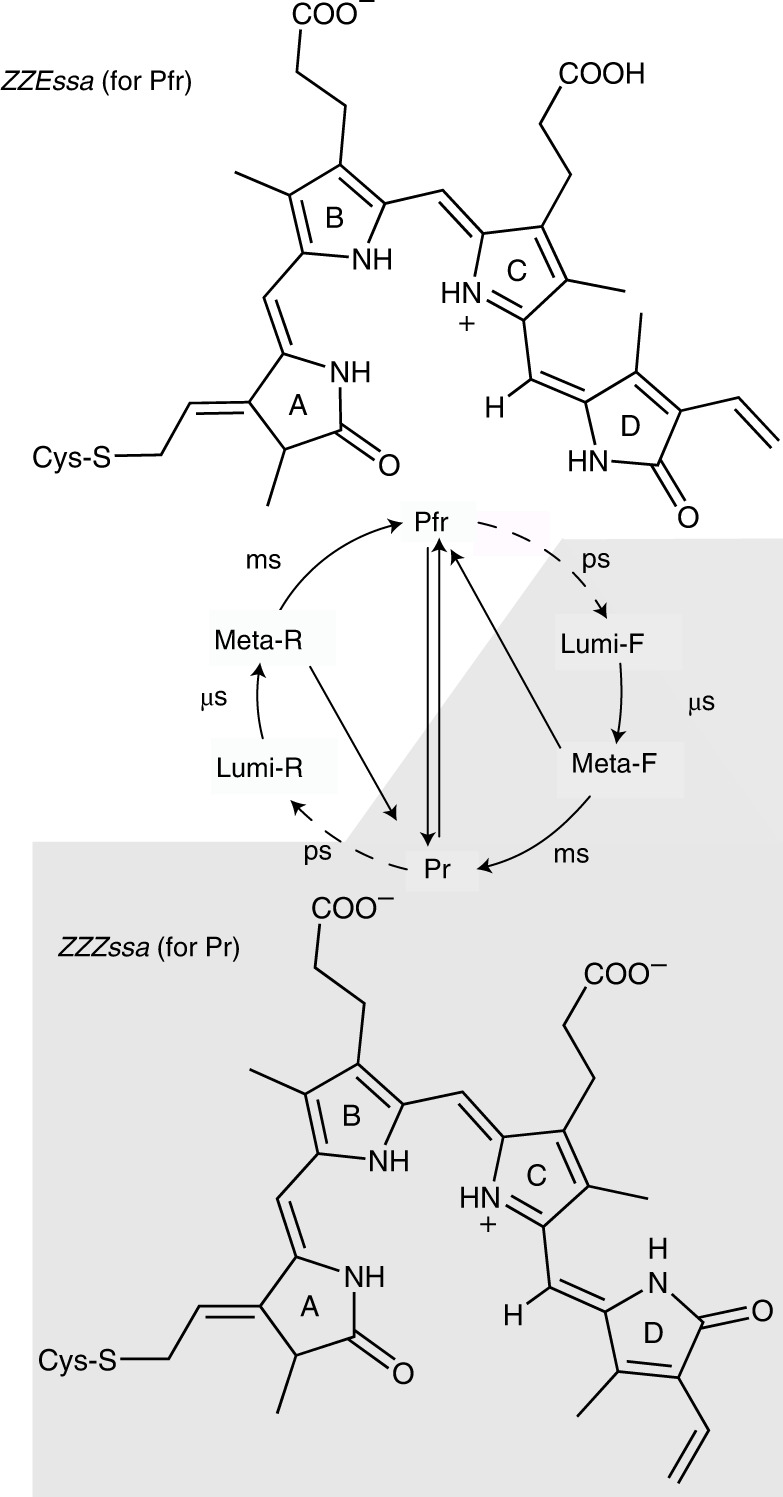
Photocycle of bacterial phytochromes as derived from spectroscopic data^28^. In Agp2, Pfr is the stable dark state. The biliverdin chromophore (BV) is bound to a cysteine residue, with a *ZZZssa* and *ZZEssa* configuration in Pr and Pfr, respectively. In both states, the chromophore is protonated at all four pyrrole nitrogens. The curved solid and dotted arrows refer to thermal and photochemical reactions, respectively. Upon photoexcitation, isomerization at the methine bridge between rings *C* and *D* occurs on a picosecond timescale, corresponding to a *ZZZssa* → *ZZEssa* and *ZZEssa* → *ZZZssa* conversion between Pr and Pfr, respectively. The last step in the reaction cascade from Pfr to Pr is associated with a structural change of the tongue segment in the PHY domain (depicted in Fig. 2), a phytochrome-specific building block of the photosensor. States highlighted by the white and grey backgrounds represent the BV chromophore in the *ZZZssa* and *ZZEssa* states, respectively. ms, millisecond; ps, picosecond; μs, microsecond.

Phytochromes play a key role in the photomorphogenesis of plants, thereby inducing physiological processes such as flowering, shade avoidance and seed germination. These photoreceptors are also found in cyanobacteria, bacteria and fungi, but their respective biological functions are only known in a few cases^1–5^. Common to all phytochromes is the photoconversion between two parent states absorbing in the red and the far-red spectral range, named Pr and Pfr, respectively. The light-absorbing cofactor is a linear tetrapyrrole covalently attached to a Cys residue via ring *A*, with a *ZZZssa* and *ZZEssa* configuration in Pr and Pfr, respectively (Fig. 1)^6–21^. In this Article we study the bathyphytochrome Agp2 from *Agrobacterium fabrum*, with a thermodynamically stable Pfr state and determined crystal structures of Pfr and the Meta-F state^22–24^.

Together with vibrational spectroscopy studies^25–28^, it was thus possible to identify key structural elements in the chromophore binding pocket that are essential for communicating the light-induced structural changes of the chromophore to the tongue segment of the protein^29^. These are, inter alia, residues Y165, R211 and His278 and the propionic side chains *B* (prop*B*) and *C* (prop*C*)^17,25,27,28,30^. Specifically, prop*C* plays a remarkable role as it is protonated in Pfr but deprotonates with the decay of the Meta-F as a prerequisite for the structural conversion of the tongue. In conjunction with the structures determined for the Pr state of prototypical phytochromes^6–8,31^, these data provide a gross picture about the main structural changes of the chromophore and the protein during the Pfr → Pr conversion.

However, the concatenation of the individual reaction steps, including chromophore isomerization and relaxation^14–16,20,32,33^, structural rearrangement of the immediate protein environment^34^, proton transfer^9,16,20,35,36^ and the secondary structure transition of the tongue^17,29,30,37^, is far from fully understood.

In this Article we study the relationship between these processes and the first primary events following electronic excitation of the chromophore in the Pfr state. We demonstrate two reaction pathways: an ultrafast reaction to the ground state and an excited-state proton transfer from the chromophore’s ring *D* to a water network in the chromophore binding pocket, stabilized by through-space interaction with the induced electric field at the chromophore. The proton-loaded water network is traced by a broad continuum band (CB) that interacts with specific side groups, such as H278. These side groups have a strong impact on the reaction dynamics demonstrated by pH dependence and site-specific mutations. With the decay of the excited state, the CB decays and the early Lumi-F photoproduct (ELF) is formed. ELF differs in hydrogen bonding from later Lumi-F sub-states, which is interpreted as a trigger for ongoing structural changes during the photocycle.

## Results

### Quantum mechanics/molecular mechanics calculations

**Fig. 2:**
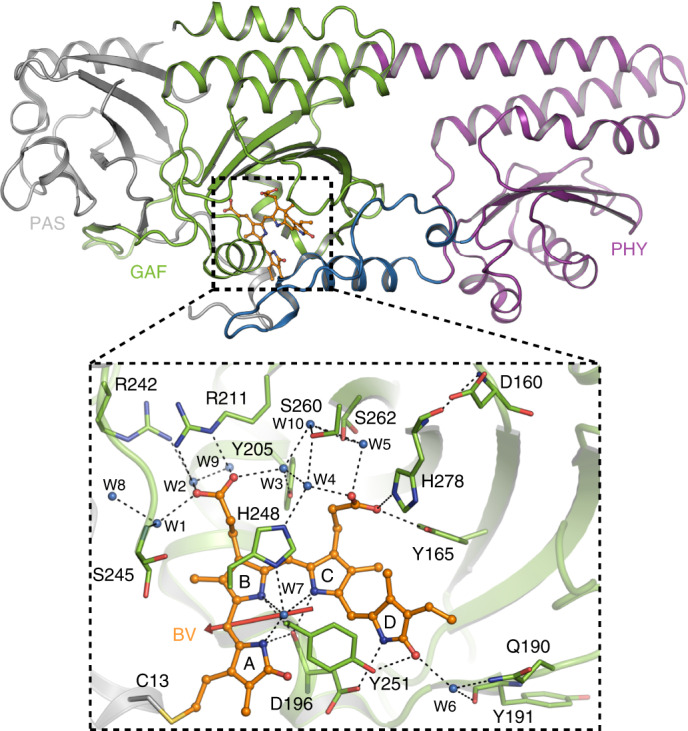
Crystal structure of Pfr Agp2. The whole structure (PDB 6G1Y) (upper panel) with PAS (grey), GAF (green), and PHY (purple) domains and the tongue region (blue), and a close-up view in the chromophore binding region around the BV chromophore (lower panel). The BV is covalently linked to the C13 side chain via ring *A*, and stabilized by various intermolecular interactions including hydrogen bonds with the side chains of Y165, Q190, D196, R211, R242, H248, Y251 and H278 as well as the water molecules W3, W4, W6 and W7. As a result, BV is embedded in a complex hydrogen-bonding network. Prop*B* forms a salt bridge to R211; prop*C* is hydrogen-bonded to Y165 and H278 and is connected via a hydrogen-bond water network (HBWN) with prop*B*; water molecules W1 to W10. The electronic transition dipole moment of the chromophore (Extended Data Fig. 1) is indicated by the red arrow. D196 is hydrogen-bonded to the NH group of ring *D*, and its backbone C=O group is hydrogen-bonded to the NH groups of rings *A*, *B* and *C*, as well as to the conserved water W7. Rotation of ring *D* takes place upon photoexcitation. Note that the water molecules are included in the original PDB entry, but not with the same numbering.

**Extended Data Fig. 1:**
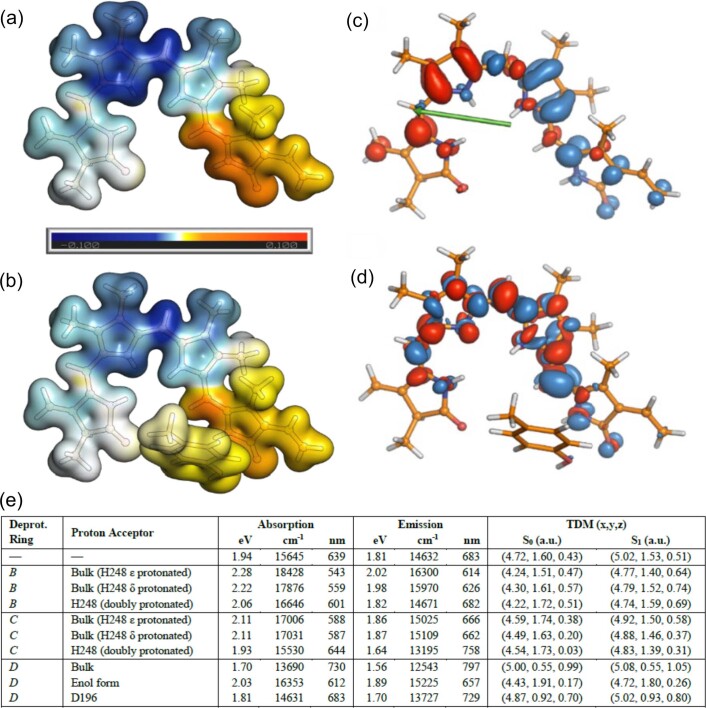
QM/MM approach for electronic transitions. QM/MM approach for electronic transitions. Excitation energies for the S_0_ → S_1_ transitions were computed using RI-CC2/cc-pVDZ for different models with protonated and deprotonated pyrrole rings by transferring the proton to the bulk or to adjacent amino acid side chains (see (f)). The model with a fully protonated BV chromophore is consistent with the Pfr ground state and has an S_0_ → S_1_ excitation energy of 15645 cm^−1^, 2347 cm^−1^ higher than the experimentally measured absorption maximum. Steady state fluorescence spectra of Agp2-WT are not available due to its ultrafast excited state deactivation. Calculated transition energies differ slightly from the experimental ones, but relative transition energies differences are more precise. Thus, we compare spectral shifts. We transferred the proton from ring *B*, *C*, and *D* to bulk water, to H248 or D196, or changed the protonation state of H248 from ε to δ (see (f)). (a) and (b): Difference in electrostatic potential between first excited state and ground state of BV in Pfr. Upon excitation the electron density increases at ring B (blue color). Negative values (blue) indicate a decrease when going from the ground to the first excited state, while positive values (red) indicate an increase. The electrostatic potential is mapped onto the electron density at an isovalue of 0.02. (**a**) Difference in electrostatic potential with only BV (excluding propionates) inside the QM region. (**b**) Same as in (a) but with Y251. For both QM regions the potential increases near the *D* ring (bottom right) and decreases near the *B* ring (upper left). c) and d): Density differences between first excited state and ground state of BV in Pfr. Negative values (blue), positive values (red, the density difference is visualised for an isovalue of ±0.001). (**c**) Transition density with transition dipole moment (TDM) vector for the S_0_ → S_1_ transition. The TDM(x,y,z)=(4.72, 1.60, 0.43) a.u. (**d**) Electron density difference (EDD) between first excited state and ground state of BV in Pfr. The EDD was computed with CC2/cc-pVDZ and shows how the electron density shifts upon excitation from the ground (blue) to the first excited state (red). Changes in the EDD are mostly located on the *B* and *C* ring of BV. Contribution of Y251 to the excitation is negligible. There are also other tyrosines, for example Y165, but the electronic TDM of these tyrosines are located perpendicular to the TDM of BV, excluding an efficient coupling. (**e**) Table: Excitation from the ground state (S_0_) to the first excited state (S_1_) for S_0_- and S_1_-optimised structures (absorption and emission, respectively), as well as S_0_ and S_1_ transition dipole moments (TDM).

The S_0_ → S_1_ excitation of the biliverdin (BV) chromophore creates an altered charge distribution in the electronic excited state (ES), connected with the electronic transition dipole moment (TDM) vector (Methods and Fig. 2, red arrow). Upon excitation, the electron density is increased at ring *B* and decreased at the methine bridge between rings *C* and *D* (Extended Data Fig. 1). Deprotonation of the chromophore is expected to affect the spectral position of the stimulated emission. The relative effect on the spectral position upon deprotonation of the chromophore is calculated for different scenarios (Extended Data Fig. 1). Redshifted emission compared to Pfr is found for deprotonation of ring *C* with a doubly protonated H248 (1,437 cm^−1^), deprotonation of ring *D* with a protonated D196 (905 cm^−1^) and for deprotonation of ring *D* with a proton transferred to the (water) bulk (2,089 cm^−1^).

### Electronic dynamics of the Pfr state

**Fig. 3:**
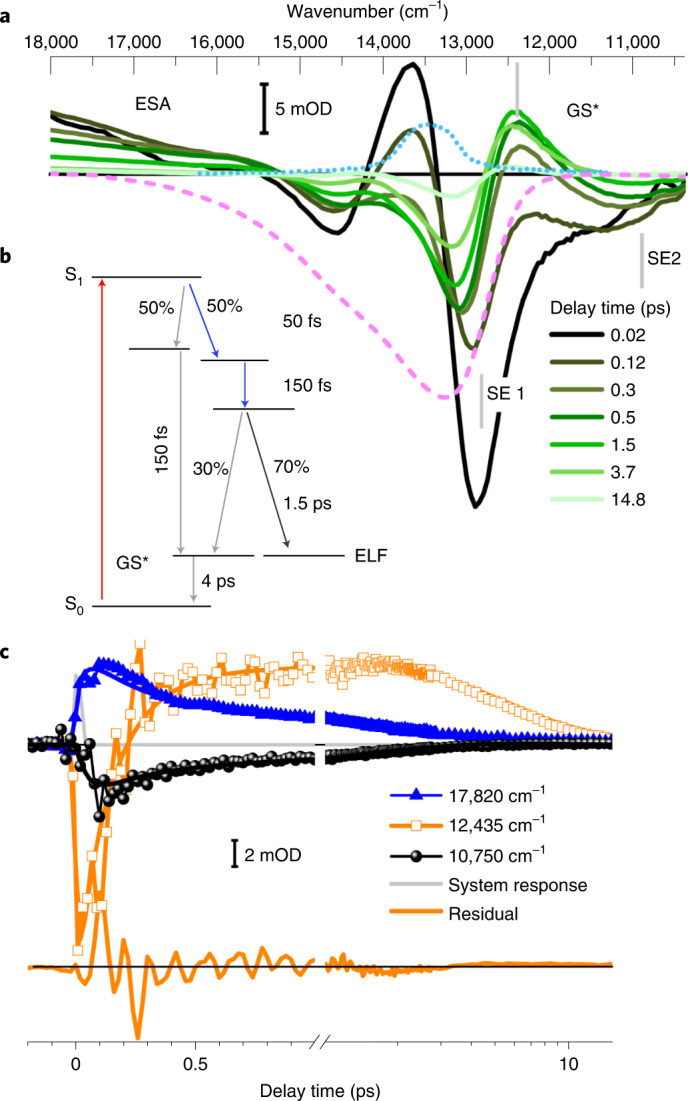
Wild-type Agp2 Pfr dynamics at pH 7.5 with excitation at 742 nm, dynamics as a function of wavenumber and pump–probe delay times. Isotropic signals reflect bleaching (−), stimulated emission (−), excited state (+) or product absorption (+); system response < 40 fs full-width at half-maximum (FWHM). **a**, Absorption difference spectra at selected delay times (colour key), pump pulse spectrum (cyan dotted line), bleaching signals following the inverted absorption spectrum (pink dashed line) from 12,000 cm^−1^ to 17,000 cm^−1^, ES absorption (ESA) from 18,000 to 13,500 cm^−1^ and SE signals below ~13,000 cm^−1^. At frequencies above 17,000 cm^−1^ and below 12,000 cm^−1^ we see a complete decay of the ESA and SE on a timescale of 3.5 ps. Dynamics after this timescale represent ground-state processes. The centre positions of SE1, SE2 and GS* are indicated by grey bars. Absorption signal changes in mOD, i.e. 10^−^^3^. **b﻿**, Proposed reaction scheme: excitation to S_1_ (red arrow); relaxation and 1:1 bifurcation in the ES with 50 fs to protonated (grey arrows) and deprotonated (blue arrows) chromophores; 150-fs relaxation of the protonated fraction to GS* and relaxation of the deprotonated fraction in the ES; reprotonation of the chromophores and decay to ELF (70%, black arrow with isomerization) and to GS* (30%) with 1.5 ps; GS* decay with 4 ps to S_0_. The overall quantum yield (QY) of ELF generation is 35%. **c**, Transients at selected wavenumbers display ultrafast dynamics in the electronic ES (triangles) and the dynamics of the SE (squares and circles). Simulated dynamics (lines) are shown with decay times of 50 fs, 150 fs, 1.5 ps and 4 ps. The solvent signal (grey) reflects the system response. The 4-ps dynamics visible in **c** (squares) reflect cooling and relaxation of the ground state GS* to the parent Pfr state. ‘Residual’ indicates the difference between the data and simulations at 12,435 cm^−1^ (orange line), displaying coherent oscillations up to ~2 ps. Source data

**Extended Data Fig. 2:**
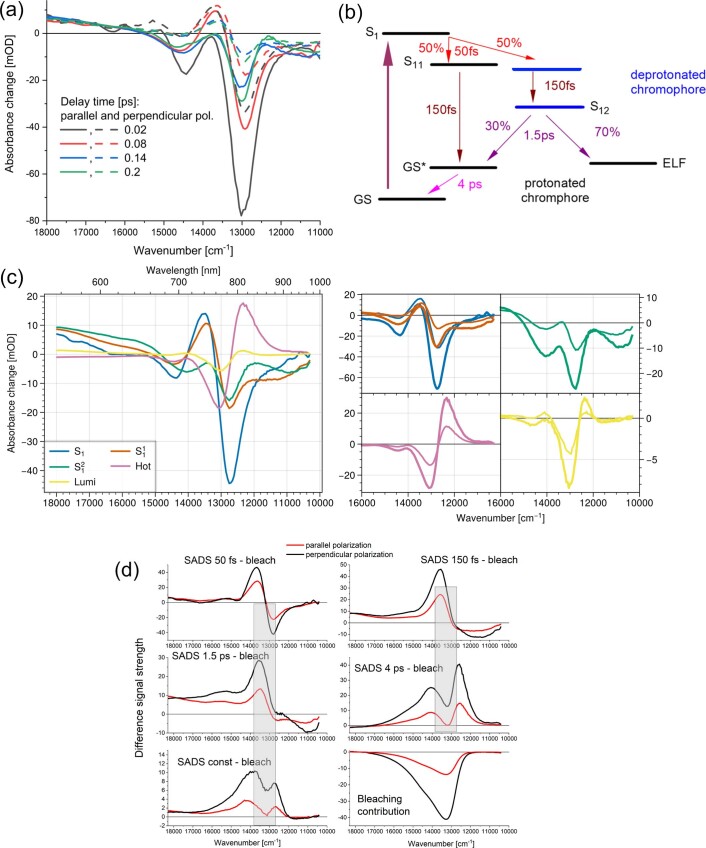
Polarization resolved data and analysis. Polarization resolved data and analysis. Agp2-WT at pH 7.5, excited at 740 nm. (**a**) Absorption difference spectra; parallel (solid lines) and perpendicular polarized (dashed lines) with respect to the pump pulse polarization; around 13700 cm^−1^ parallel and perpendicular signals deviate for small delay times and at 200 fs, but are nearly identical at 80 fs; at 13000 cm^−1^ parallel and perpendicular signals show similar ratios (~2) for parallel and perpendicular polarization for all presented delay times; around 17500 cm^−1^ parallel and perpendicular polarizations show nearly identical signals. (**b**) Target model from Fig. 3b used to explain the observed dynamics. Here, we introduced two apparent states S_11_ and S_12_ for clarification. Two yields, QY_1_ and QY_2_ had to be estimated. The formation of the deprotonated chromophore in the electronic excited state S_11_ (blue line) is given by QY_1_ = 0.5, and the formation of the protonated photoproduct ELF is given by QY_2_. To do the estimation, we looked at the polarization resolved signals after subtraction of the bleaching contribution (d). Next, we assumed that the excited state absorption in the S_11_ represents two electronic contributions, a protonated one (black line) and a deprotonated one (blue line); the S_12_ electronic state consists of a single electronic transition (deprotonated); the anisotropy of S_11_ and S_12_ is very similar around 15000 cm^−1^ indicating similar transition dipole moments (see c and d). The modelling was best given for QY_1_ ~ 0.5 and QY_2_ ~ 0.7, resulting in a total early Lumi-F yield of around 0.35. The value is in good agreement with the estimation from our IR data comparing the initial and final amplitude of the ring *D* v(C = O) mode (Fig. 4a lower inset). (**c**) Species associated difference spectra (SADS) from the target mode in (c): Left panel: isotropic SADS spectra; Right panel: polarization resolved SADS, parallel (thicker lines) and perpendicular polarization (thinner lines), with the same color code for both panels. Nomenclature used from (b). (**d**) Polarization resolved SADS spectra subtracted by the bleaching contribution for the target model in (b), and the polarization resolved bleaching contribution upon photoexcitation; S_1_: SADS 50 fs - bleach, S_11_: SADS 150 fs - bleach, S_12_: SADS 1.5 ps – bleach; GS*: SADS 4 ps – bleach, and ELF: SADS const - bleach. The fraction of the bleaching contribution follows the target model and quantum yields. The spectral region with influence from scattering light (increasing with decreasing signal strength) is marked by a grey rectangle. Source data

**Extended Data Fig. 3:**
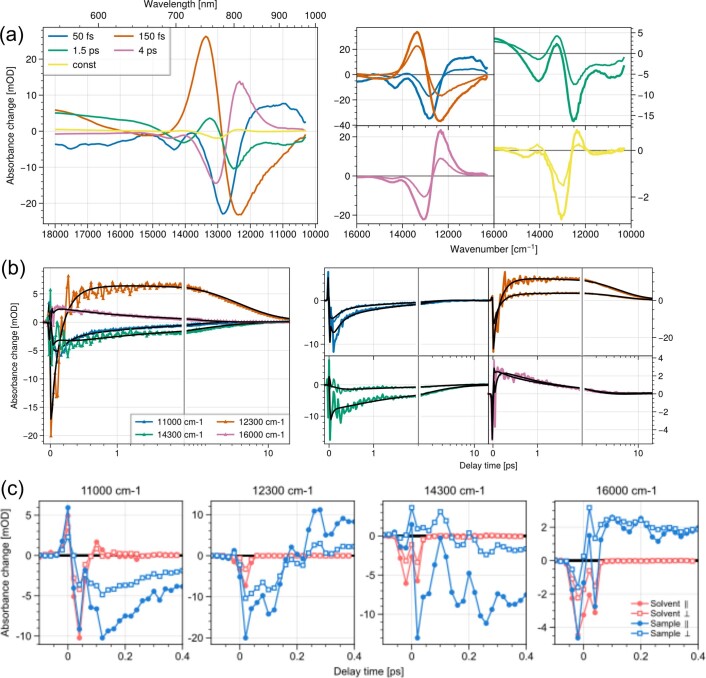
Dynamics in the visible range. Dynamics in the visible range. (**a**) Decay associated spectra of Agp2-WT at pH 7.5 excited at 740 nm (40 nm FWHM, < 40 fs time-resolution). Left panel shows the isotropic decay associated spectra. The right panels show the same components polarization resolved. Thicker lines present parallel polarization of pump and probe, thinner lines the perpendicular polarized case. (**b**) Transients of the same dataset; data (colored) and resulting model (black) for selected frequencies. Left panel shows isotropic data and simulations, while the right panel shows the polarization resolved data. Thick lines are for parallel polarization, thin lines for perpendicular polarization. Used decay constants are 50 fs, 150 fs, 1.5 ps, 4 ps and a constant component. For the very early delay times small deviations between modelled transients and data are visible. Introducing an additional time constant around 100 fs would have resulted in reduced deviations, but also in an ill-conditioned model. (**c**) Comparison of the solvent signal (red) with the sample signal (blue) at different spectral positions and different polarization directions with respect to pump pulse polarization. Source data

Polarization-resolved femtosecond VIS-pump broadband VIS-probe experiments were performed to identify the photoreaction dynamics of Pfr (Fig. 3). In Fig. 3a we see the very early absorbance difference spectrum at 20 fs after excitation (black line). On this timescale the stimulated emission (SE) band develops and shifts from ~12,800 (black spectrum, 20-fs delay) to 11,000 cm^−1^ at 0.5 ps (green spectrum). This redshift of SE is only consistent with our calculations for a deprotonated chromophore at ring *D* or at ring *C* (Extended Data Fig. 1). The strongest signal changes are observed within 300 fs—with time constants of 50 fs and 150 fs we observe a decay of SE1 (centred around 12,900 cm^−1^) and with a time constant of 50 fs the rise of the redshifted SE2 around 11,000 cm^−1^. Thus, we assign this fast 50-fs process to the deprotonation of the chromophore in the ES (Fig. 3b). This change in electronic states is reflected by distinct ES anisotropies of the species-associated difference spectra at 50 fs and 150 fs (Extended Data Figs. 2 and 3). Further processes on the ES potential energy surface (PES) occur with time constants of 150 fs and 1.5 ps.

Our data support a branching of the population on the ES PES^19^. One fraction of the ES population on the PES decays with a time constant of 150 fs and the other fraction on the PES decays in 1.5 ps (Fig. 3b). The timing of the branching occurs concomitant with the early relaxation of ~50 fs, separating the protonated and deprotonated pathway (Fig. 3b). The yield for this ultrafast branching process on the ES PES is modelled with QY_1_ = 0.5 (QY, quantum yield; Fig. 3b).

**Extended Data Fig. 4:**
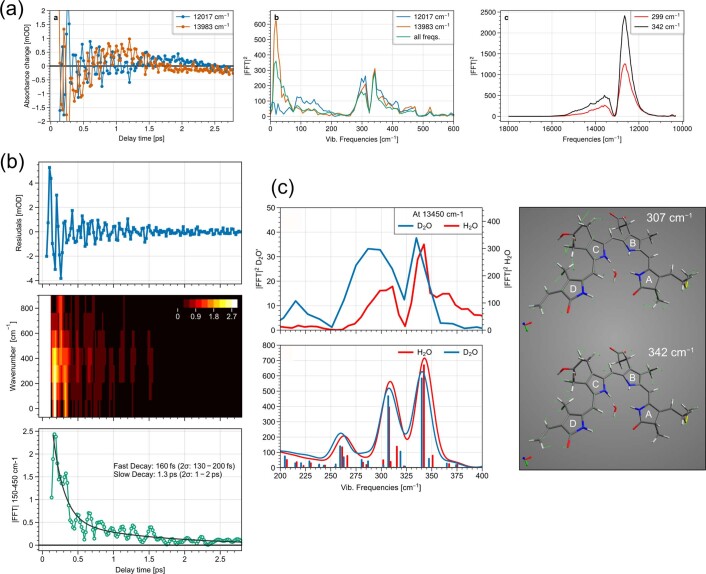
Coherent oscillations in Agp2-WT at pH 7.5. Coherent oscillations in Agp2-WT at pH 7.5. (**a**) Left: Residuals after subtraction of the exponential model. The coherent oscillations are clearly visible. The shown oscillations show a relative phase-shift of π; middle: The resulting power-spectrum. The main features are two peaks at ~304 and ~340 cm^−1^; right: The power of the two frequencies at 299 cm^−1^ (red line) and 342 cm^−1^ (black line) as a function of the probed visible frequency. (**b**) Top: Residuals at 14200 cm^−1^ after applying a running mean. Middle: STFT of the residuals. The color indicates the Fourier-amplitude (not power). The STFT used a 6 points per segment with 5 overlapping points and applied a Hann-window. Clearly visible is the high average amplitude from 150 to 450 cm^−1^ as a function of time. Bottom: Most of the amplitude decays within several hundred femtoseconds. The remaining amplitude decays on a timescale of about 1 ps. The black line presents a two-exponential fit of the amplitude, with the first point ignored. (**c**) Upper left: Power-spectrum at 13450 cm^−1^ in H_2_O (pH 7.5) and D_2_O (pD 7.8). Lower left: Calculated Raman-spectra for deuterated and protonated pyrrole rings and prop*C*. The calculated spectrum reproduces the data rather well and lets us assign the two main peaks to specific normal modes at 307 cm^−1^ and 342 cm^−1^ (right panel). Both involve ring *D* rocking and out-of-plane bending of the methine-bridge between ring *C* and *D*. Source data

Halving of the ES signal at 14,700 cm^−1^ and SE1 at 12,800 cm^−1^ within 120 fs can be explained by the concomitant rise of a positive band at 12,435 cm^−1^ with a time constant of 150 fs. This positive band decays with a 4-ps time constant, longer than the ES decay time constants of 150 fs and 1.5 ps (Extended Data Fig. 3). Hence, we assign this positive signal to a hot ground-state absorption GS* crossing the ES PES via a conical intersection. The photoreaction is accompanied by strong coherent oscillations, as depicted in Fig. 3c (orange line) (Extended Data Fig. 4). These coherent oscillations decay with time constants of 160 ± 40 fs and 1.5 ± 0.5 ps (Extended Data Fig. 4). The decrease in intensity of the coherent oscillations with 150 fs supports the existence of a conical intersection in the ES PES. We assign the dominant coherent oscillations at 300 and 340 cm^−1^ to out-of-plane vibrations of the chromophore’s ring *C* and *D* (Extended Data Fig. 4). Because the calculated Raman intensities are stronger for the protonated chromophore, we assign the coherent oscillations to the reaction pathway with a fully protonated chromophore.

**Fig. 4:**
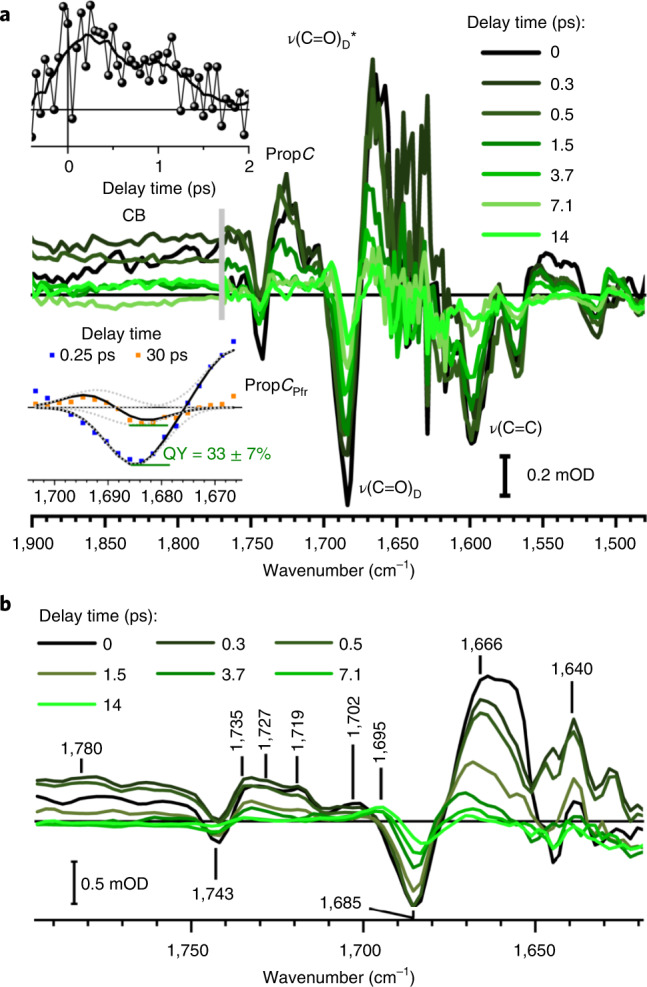
Vibrational absorption difference spectra of Agp2-WT at different delay times after photoexcitation in D_2_O. **a**, Spectral region from 1,900 to 1,480 cm^−1^ at pD 8.2. Two datasets are separated by a grey bar: an isotropic dataset from 1,770 to 1,480 cm^−1^ and a scaled perpendicular polarized dataset from 1,900 to 1,770 cm^−1^. Strong sample absorption increased the noise around 1,640 cm^−1^. CB indicates the proton-loaded water network; prop*C*_Pfr_ and prop*C* indicate the carbonyl vibration of prop*C* in the Pfr ground state and after photoexcitation, respectively. *v*(C=O)_D_ and *v*(C=O)_D_* correspond to the bleaching signal and ES of carbonyl ring *D*, respectively. *v*(C=C) represents the vibrations of *A*–*B* stretching and ring *B* stretching. The spectral range from 1,600 to 1,520 cm^−1^ shows mainly C=C stretching dynamics of the chromophore with strong bleaching signals at 1,596 cm^−1^ (*A*–*B* stretching) and 1,566 cm^−1^ (ring *B* stretching), accompanied by redshifted positive signals due to excited-state and hot ground-state absorption. Upper inset: transient of the presented CB averaged from 1,772 to 1,832 cm^−1^ and the smoothed transient (black line). Lower inset: QY estimation by comparing signals at the beginning (blue squares) and after photoreaction (orange squares). Simulations for *v*(C=O)_D_ at 1,684 cm^−1^, *v*(C=O)_D_* at 1,666 cm^−1^ at the beginning and *v*(C=O)_D_ at 1,684 cm^−^^1^ and *v*(C=O)_DELF_ at 1,692 cm^−1^ after photoreaction. Individual bands (dotted lines) and sum of bands (black lines). The QY of 33 ± 7% is given by the ratio of the two *v*(C=O)_D_ signals. **b**, Isotropic dataset from 1,795 to 1,619 cm^−1^ at pD 9. Peak positions are indicated. Two small peaks at 1,719 and 1,702 cm^−1^ are indicated in the first 0.5 ps. In this spectral range, signals from a protonated D196 would be expected. Interestingly, the carbonyl stretching of prop*C* shows a frequency downshift in the ELF upon chromophore excitation, although prop*C* is not directly connected to the chromophore’s delocalized *π*-electron system. However, deprotonation of ring *D* or ring *C* may cause subtle alterations of the structural and electronic properties of the propionic side chain, as also suggested by the calculated frequencies (Extended Data Fig. 7). Note that such effects may account for the RR activity of this mode (Extended Data Fig. 10). Source data

**Extended Data Fig. 5:**
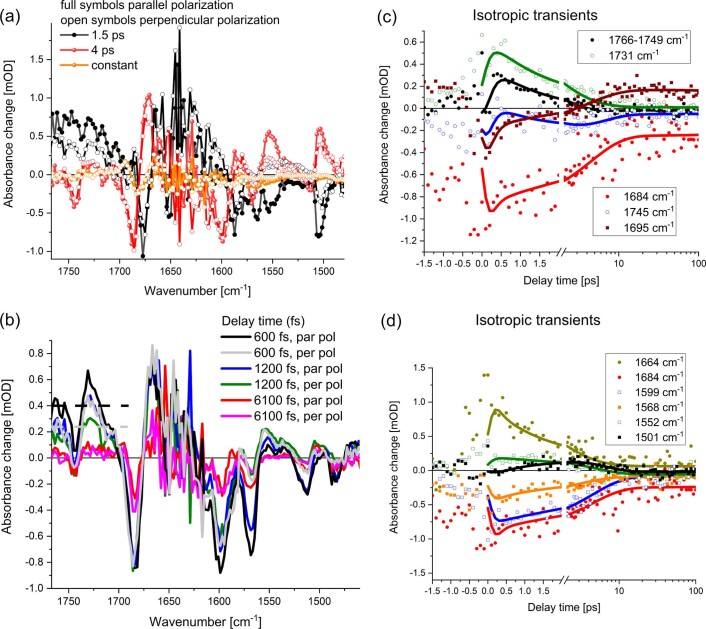
Polarization resolved and isotropic vibrational dynamics. Polarization resolved and isotropic vibrational dynamics. (**a**) Polarization resolved decay associated spectra of Agp2-WT at pD 8.2; parallel polarization (full symbols), perpendicular polarization (open symbols). The dataset was fitted with fixed decay constants of 150 fs, 1.5 ps, and 4 ps for both datasets simultaneously. The 150 fs components are not shown, because of strong mixing with the non-linear artefact during the system response. Comparing the DAS signals for 1.5 ps and 4 ps in the spectral range from 1600 cm^−1^ to 1480 cm^−1^ show similar or slightly red-shifted negative and positive signals. This supports the assignment of a hot ground state to the 4 ps dynamics. The origin of the CB can be further investigated by its polarization resolved signals. After photoselection by our linear polarized pump pulse we compared the absorption signals of parallel (A_par_) and perpendicular (A_per_) polarized probe pulses with respect to the pump pulse polarization. Taking the dichroic ratio D = A_par_/A_per_ from 1780 to 1751 cm^−1^ and simulating the data with a horizontal line, we found D = 1.7 ± 0.3 corresponding to a relative angle between the TDM of the CB band and the S_0_ → S_1_ TDM (see Fig. 2) of 30° to 43° or (34 ± 10)°. The expected vibrational TDM for a CB is almost completely polarized along the direction of maximal hydrogen bonded water network extension, supported by our ab initio Born-Oppenheimer MD simulations (see Extended Data Fig. 6). (**b**) Polarization resolved absorption difference spectra for different pump-probe delay times of Agp2-WT at pD 8.2 with delay times averaged at 600 fs (500 to 700 fs), 1200 fs (900 to 1300 fs), and 6100 fs (5500 to 6800 fs); an adjacent averaging filter (3 points) was used to smooth spectra. The spectra from 1660 cm^−1^ to 1780 cm^−1^ and 1490 to 1620 cm^−1^ are from two different experiments. The dotted black and grey lines indicate the level of the CB for the absorption difference spectra at 600 fs for parallel and perpendicular polarization. From 1780 to 1751 cm^−1^ we found D = 1.9 ± 0.3 corresponding to a relative angle of 26° to 38°. (**c**) and (**d**) Isotropic polarized transients at selected wavenumbers (open and solid symbols) together with the simulated data (solid lines) from the DAS presented in (a). After about 10 ps the photoreaction is completed and the first photoproduct early Lumi-F (ELF) is formed. The marker band for ELF is presented at 1695 cm^−1^. Note, the perturbed free induction decay (PFID) is responsible for non-zero signals before time zero. Proper simulations of PFID signals are very demanding for a multitude of contributing bands in adjacent spectral regions. Source data

The deprotonated fraction of the ES population develops on the PES with increasing rotation of ring *D*^10^ and decays with a time of 1.5 ps (Fig. 3c, circles and triangles) to the isomerized photoproduct state ELF and the ground state GS* (Extended Data Fig. 2). The total ELF generation yield of ~0.35 is taken from the time-resolved vibrational measurements depicted in Fig. 4a, lower inset. Our vibrational data show very similar frequencies in the decay-associated spectra for negative and positive bands between 1,600 and 1,500 cm^−1^, supporting a protonated chromophore in GS* and in ELF and indicating a reprotonation of the chromophore with the isomerization of ring *D* and transition to the ELF or GS* (Extended Data Fig. 5).

Accordingly, we interpret the ultrafast dynamics of Agp2-WT as an excited-state proton transfer from the chromophore with a time constant of ~50 fs to a protein acceptor group, with a subsequent reprotonation and isomerization of the chromophore and photoproduct formation of ELF on a timescale of 1.5 ps. The chromophore acts as a proton donor in its electronic excited state.

### Transient proton transfer and vibrational dynamics

**Fig. 5:**
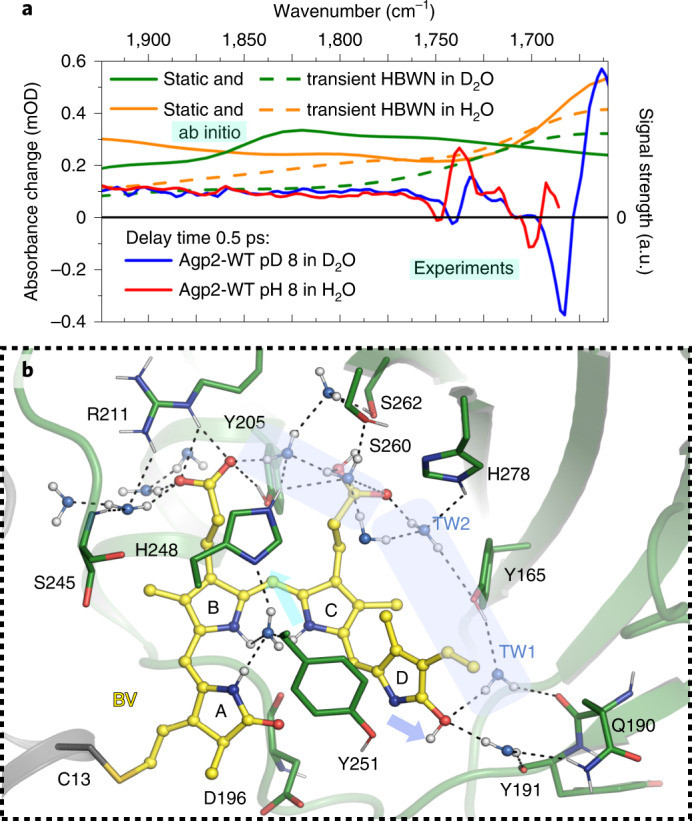
Water networks in Agp2-WT. **a**, Absorbance difference spectra of Agp2-WT in H_2_O (red) and in D_2_O (blue) at 0.5 ps (averaged from 0.4 to 0.6 ps) after photoexcitation at 765 nm, compared with calculated absorption spectra (scaled) of two different proton-loaded HBWNs in a confinement in H_2_O (orange) and D_2_O (green) by ab initio Born–Oppenheimer MD simulations (Extended Data Fig. 6): static HBWN between prop*B* and prop*C*; transient HBWN between prop*C* and W6. **b**, Structural snapshot from MD simulations up to 10 ns performed on Agp2-WT with an enolic ground state. A metastable transient water network (blue area) is found. Hydrogens of water molecules and hydrogen bonds are shown. Potential transient waters TW1 and TW2 were identified in the crystal structure (PDB 6G1Z) with a lower electron density compared to other water molecules published in the crystal structure. The potential transient waters presented here were not published in the PAiRFP2-Pfr crystal structure (PDB 6G1Z), because their electron density is lower compared to the conventional limit for interpretable electron densities of water molecules. The lower electron density in the X-ray structure could reflect a lower residence time and higher flexibility, that is, transient waters, in the ground-state structure, supported by the metastable geometry in MD simulations. The transient water network extends from the CO group of ring *D* via TW1, Y165, TW2, H278 to prop*C* and prop*B*. Blue and cyan arrows indicate possible proton transfer pathways from ring *D* to TW1 and from ring *C* to H248, respectively. Source data

**Extended Data Fig. 6:**
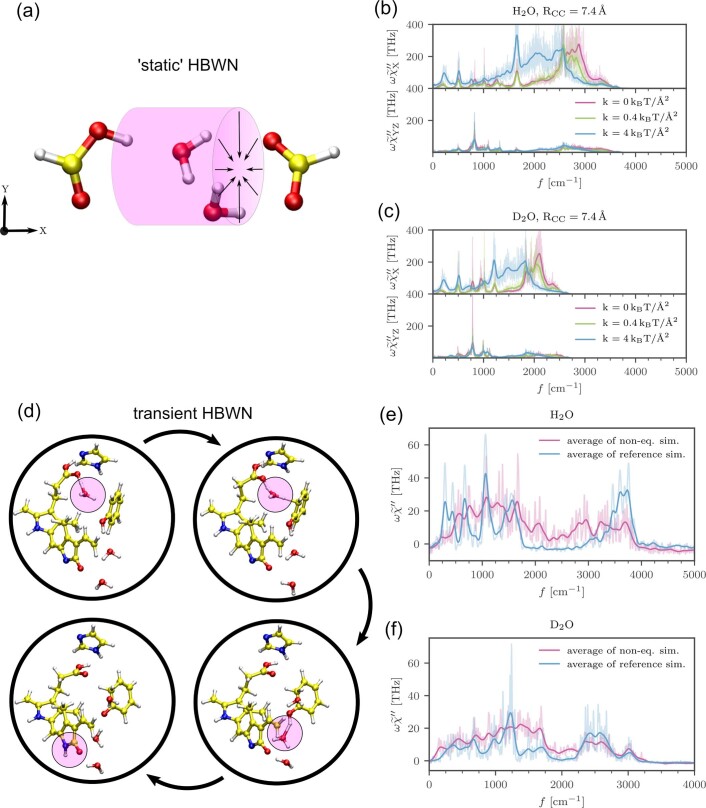
Ab initio Born-Oppenheimer MD simulations. Ab initio Born-Oppenheimer MD simulations. (**a**) A hydrogen bonded water network (HBWN) taken from the ground-state structure of Agp2 of two water molecules in between a pair of carboxylic acid molecules. One carboxylic molecule is deprotonated. The total charge is -1e. The pink cylinder illustrates the confining cylindrical potential of strength k acting on the water oxygen nuclei. The carbon atoms are constraint at distance R_CC_, as suggested by the crystal structure of Agp2. (**b**, **c**) IR spectra along different axes for different confining strength k in k_B_T/Å^2^ for H_2_O (b) and D_2_O (c). All systems show a much higher IR intensity along the X axis than in the YZ plane. The signals in the YZ plane are mostly independent of the confining strengths k. For both, H_2_O and D_2_O, clear bands reside at 500, 800 and around 1000 to 1200 cm^−1^ that are associated with the modulated C-O vibrations. In addition, a continuum band appears between 2500 cm^−1^ and 3500 cm^−1^ for H_2_O and between 1500 cm^−1^ and 2500 cm^−1^ for D_2_O. The relatively stronger signal along the X axis on the other hand heavily depends on the confining strength k. Excess proton exchange between two carboxylic acid molecules occurs at the ps time scale. These model simulations reveal the effect of confinement on water-mediated proton transfer dynamics and its spectral signature, specifically the appearance of a continuum band in the 1700 cm^−1^ - 2000 cm^−1^ range for both H_2_O and D_2_O at strong confinement. (**d**) A transient HBWN, including one side of the chromophore with ring *C* and ring *D*, side chains H278 and Y165, that are each truncated at the ring, three water molecules TW1, TW2 and W6 (see Fig. 2) and an excess proton, the total charge amounts to +1e. The pink circles illustrate the location of the excess proton during the course of a non-equilibrium simulation trajectory, where the excess proton is initially placed at the water molecule TW2 near ring *C*. (**e, f**) IR spectra, averaged over three non-eq. simulations for H_2_O (e), five non-eq. simulations for D_2_O (f) and all spatial dimensions. In the reference simulations the excess proton is located near the nitrogen atom of ring *D*. The comparison clearly shows that the non-eq. simulations exhibit a continuum band between 1700 cm^−1^ to 3000 cm^−1^ for H_2_O and between 1800 cm^−1^ and 2200 cm^−1^ for D_2_O, which are not visible in the reference simulations. In summary, the ab initio MD simulations suggest the ‘static’ HBWN in (a) and the transient HBWN in (d) as plausible candidates that produce spectra in agreement with the experimentally observed transient IR spectra. Source data

To identify the proton acceptor we investigated the vibrational dynamics of Agp2 in the fingerprint region. Difference spectra in the range from 1,900 to 1,750 cm^−1^ show a broad positive CB upon excitation (Fig. 4). Its transient is presenting a rise within 300 fs and a decay within a few picoseconds (Fig. 4a, upper inset). CBs have been reported previously for charge transfer states, shared protons between carboxylic acids and protonated water networks^38–50^. Substantial spectral changes following D_2_O to H_2_O exchange have been reported previously for shared protons between carboxylic acids^51^, although we did not detect any differences between 1,900 and 1,750 cm^−1^ in our experiment (Fig. 5a). Our ab initio Born–Oppenheimer molecular dynamics (MD) simulations for a protonated network of two water molecules between two carboxylate groups (COO^−^), as well as for a transient hydrogen-bonded water network (HBWN) between prop*C* and W6 (Extended Data Fig. 6 and Fig. 5b) show similar CBs in D_2_O and H_2_O from 1,900 to 1,750 cm^−1^ (Fig. 5a). The cylindrical confinement emulates the protein (Methods and Extended Data Fig. 6). We thus assign the observed CB, in agreement with previous observations of CBs for protonated water networks or charge transfer states^38–50^, to the formation of a transient proton-loaded water network (D_2_O…D^+^…D_2_O).

The TDM of the CB was determined by polarization-resolved measurements (Extended Data Fig. 5). We found an angle to the electronic TDM of ~34 ± 10°. The expected vibrational TDM for a CB is almost completely polarized along the direction of maximal cluster extension, supported by our ab initio Born–Oppenheimer MD simulations (Extended Data Fig. 6)^43^. The measured TDM of the CB agrees with the orientation of the water molecule network between prop*B* and prop*C*, as well as with the water network between prop*C* and W6 (Fig. 5b), or their combination. Thus, the measured angle supports the assignment to a protonated water network that is localized between prop*B* and TW1. The measured water network is loaded with a proton within 300 fs, indicating a direct interaction with the chromophore (Fig. 4a, upper inset). The CB decays with the decay of the ES and formation of ELF. Given that the water network is not part of the ES, we expect the Coulomb force of the ES to affect and stabilize the transient water network.

**Extended Data Fig. 7:**
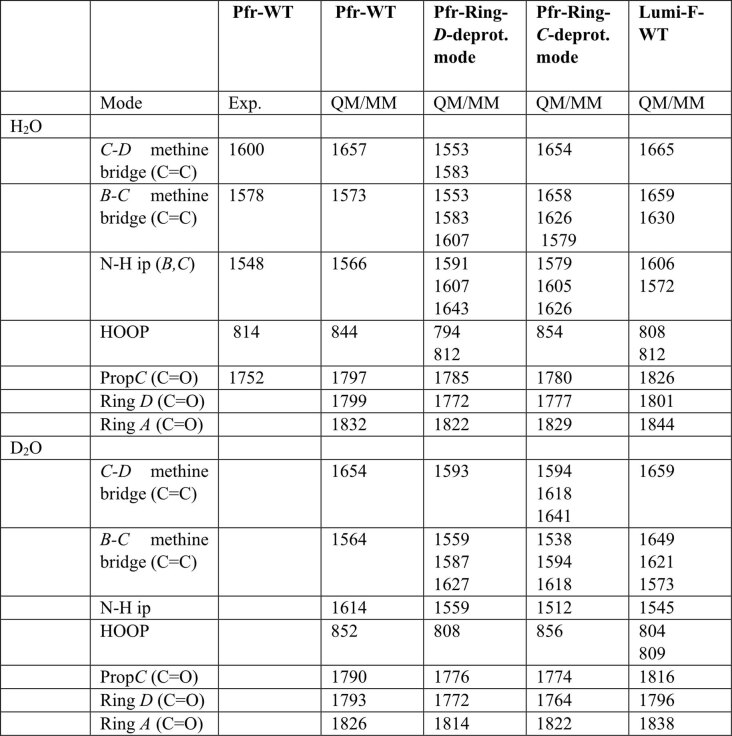
Computation of IR spectra. Computation of IR spectra. Selected QM/MM calculated (*) and experimental vibrational frequencies of vibrational modes diagnostic of the chromophore geometry for the Pfr models as well as Lumi-F model in H_2_O and D_2_O. Frequencies are given in cm^−1^. (*) The crystal structure of Agp2 in the Pfr state (PDB:6G1Y) was used as template for generating the initial structure for the Pfr and Lumi-F models. Unresolved regions in the crystal were restored by three-dimensional homology modelling using SWISS MODEL and hydrogens were added to the crystallographic structure according to predictions based on Karlsberg2 + . His248 and His278 were modeled as charge neutral each with a proton at Ne position. The Lumi-F model was generated based on the Pfr model by simply rotating the pyrrole ring *D* around 180 degrees. The chromophore binding site of the energy minimized- and thermally equilibrated solvated protein models were geometry optimized at QM/MM level. Accordingly, the biliverdin chromophore, the side chains of Cys13 and the pyrrole water were treated quantum mechanically at the B3LYP/6-31 G* level of theory while the protein matrix, solvent water and ions were described molecular mechanically using CHARMM36 force field. Relaxation during the minimization was allowed only for atoms located within a 20 Å-radius sphere centered at N22 of the BV cofactor. The charge-shifted scheme in combination with the electrostatic embedding approach was used to couple the QM and the MM region. The QM/MM optimized geometries were further used as input for subsequent frequency calculation of exclusively the QM fragment. These computations were performed at B3LYP/6-31 G* level of theory using GAUSSIAN09 following the same protocol as described previously in ref. ^79^. Scaling of force constants, normal mode analysis as well as correction of the QM Hessian matrix were performed using the programs developed in our group.

Other vibrational bands visible in Fig. 4 reflect the ultrafast dynamics of the Agp2-WT. Comparison with quantum mechanics/molecular mechanics (QM/MM) calculations allow for an assignment of these bands (Extended Data Fig. 7).

The chromophore’s carbonyl stretching vibration at ring *D* displays bleaching signals and excited-state signals at 1,685 and 1,666 cm^−1^, respectively. The photoproduct signal of ELF is detected at 1,695 cm^−1^, blueshifted with respect to the Pfr state. This vibrational frequency is indicative of an isomerized and protonated chromophore^10^. Comparison of the bleaching signals before and after photoreaction (Fig. 4a, lower inset) demonstrates a QY of ~0.35 for the photoisomerization at ring *D*^12^. The excitation also alters the properties of the carbonyl stretching vibration of the protonated prop*C* (Fig. 4b) with bleaching at 1,743 cm^−1^, a broad signal during the excited state or hot ground state around 1,727 cm^−1^, and the product prop*C* signal at 1,735 cm^−1^. Frequency calculations of the Pfr ground state indicate a redshift of the carbonyl prop*C* and carbonyl ring *D* of ~16 cm^−1^ and ~24 cm^−1^, respectively, upon deprotonation of ring *D* or ring *C* (Extended Data Fig. 7). The observed redshift of the carbonyl stretching vibration of prop*C* of 16 cm^−1^ during the electronic excited state (Fig. 4b) matches this calculated value. The carbonyl vibration of ring *D* is found experimentally at 1,685 cm^−1^. Its ES signal is visible at 1,666 cm^−1^ with a shoulder at 1,640 cm^−1^ (Fig. 4b). The shoulder could reflect the deprotonated chromophore in the ES, but the signal-to-noise is reduced in this spectral range due to strong amid I absorption. At ~15 ps, the ultrafast reaction is completed and the band pairs represent the photoproduct ELF (+) and bleaching Pfr (−) signal (Fig. 4, light green lines). The ELF state exhibits a redshifted carbonyl stretching vibration of prop*C* at 1,735 cm^−1^ with respect to the Pfr ground state at 1,743 cm^−1^, probably due to a stronger or a new hydrogen bond of the carbonyl group in ELF.

### Impact of pH on chromophore dynamics

**Fig. 6:**
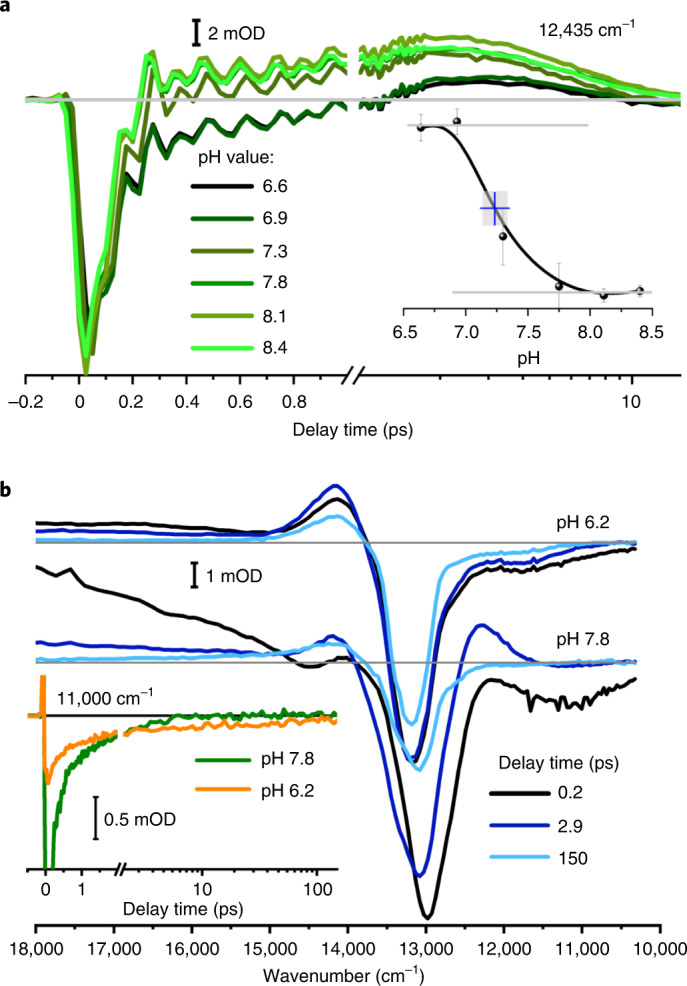
pH-dependent dynamics. **a**, pH dependence of the photoreaction of Agp2-WT at different pH values in H_2_O. All datasets were scaled at 635 nm and 1 ps. Transients are at 12,435 cm^−1^ for pH values of 6.6–8.4 (graduated line colours from black to green). Inset: pH dependence averaged from transient absorption amplitudes at fixed delay times and spectral positions. The pH dependence indicates a p*K*_a_ value of 7.2 ± 0.3. **b**, Difference spectra at selected delay times reflecting the electronic dynamics of Agp2-H278Q in H_2_O in the visible spectral range at pH 6.2 and 7.8. Inset: transients at 11,000 cm^−1^ for pH 6.2 (orange) and pH 7.8 (green). On lowering the pH from 7.8 to 6.2, the photoreaction of Agp2-H278Q is substantially slowed to ~100 ps, similar to the results for Agp2-H278A (Extended Data Fig. 9). This is reflected by the SE around 11,000 cm^−1^ at 150-ps delay time at pH 6.2. Moreover, the spectral features in Agp2-WT reflecting SE2 and GS* are missing, supporting that these features are indicative for the ultrafast photoreaction in Agp2-WT. With increasing pH starting at pH 6.2, the fraction of WT-like dynamics in Agp2-H278Q increases at the expense of the slow dynamics on the hundreds of picoseconds timescale (Extended Data Fig. 9). Source data

**Extended Data Fig. 8:**
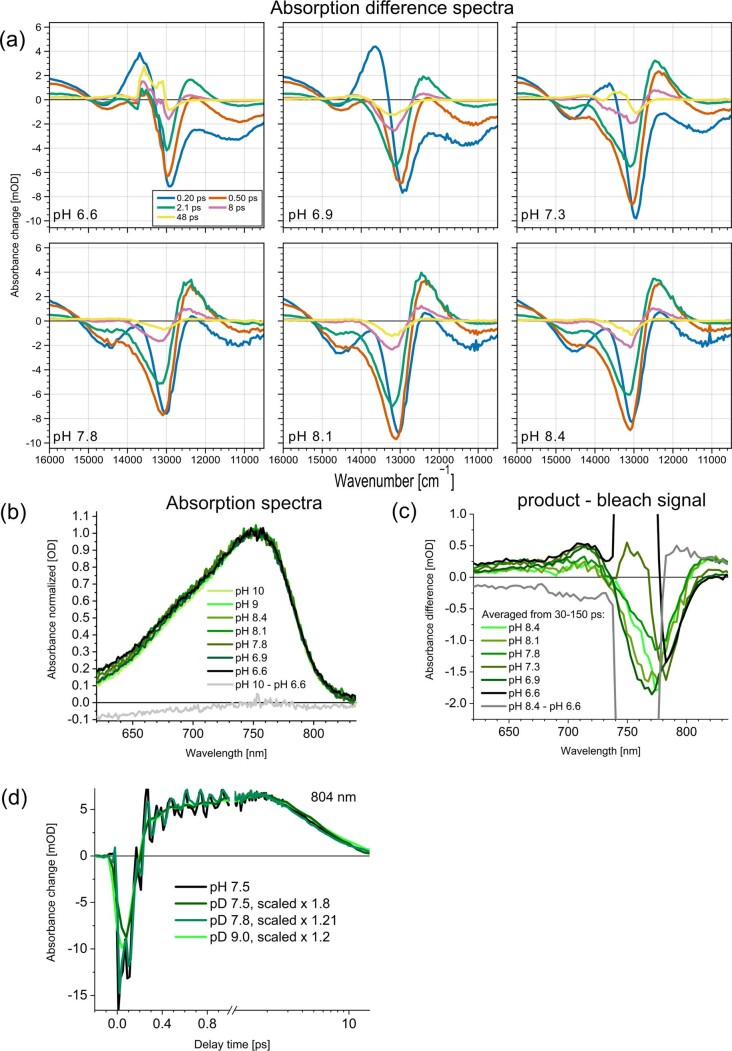
pH dependent measurements of Agp2-WT Pfr. pH dependent measurements of Agp2-WT Pfr. (**a**) Isotropic transient spectra at selected time-points of Agp2-WT excited at 750 nm at different pH values. All datasets were normalized to their signal at 635 nm at 1 ps. At lower pH, the solubility goes down and therefore increased scattering of the pump-pulse is observed around 13300 cm^−1^. While the data at higher pH values look all rather similar, the dynamic below pH 7.0 is distinctly slower. (**b**) Normalized absorption spectra of the samples measured at different pH values. The spectral difference in the Pfr state between pH 10 and pH 6.6 are small and follow the shape of the Pfr absorption spectrum. Thus, the p*K*_a_ of the Pfr chromophore is expected to be higher than 9. (**c**) pH dependence of the ELF-Pfr difference spectra averaged from 30 ps to 150 ps after photoexcitation; isotropic polarization. The different samples were measured directly after each other under the same experimental conditions. The difference spectra show contributions of early Lumi-F (positive signals) and of the Pfr bleaching contribution (negative signals); between 740 to 780 nm scattering of the pump pulse distorts the spectra (strongest at low pH values); since the Pfr shows negligible pH dependence (b) the pH dependence has to be induced by a pH dependence of the ELF absorption spectrum. The difference of pH 8.4 and pH 6.6 exhibits a sign change from 740 to 800 nm, displaying a red-shift of the ELF absorption to higher pH values. Thus, we see a pH dependence of the ELF absorption spectrum, supporting hydrogen bonding of ELF. (**d**) Direct comparison of the transients at 804 nm (12435 cm^−1^) in H_2_O at pH 7.5, in D_2_O at pD 7.5, in D_2_O at pD 7.8, and in D_2_O at pD 9.0. The transients were scaled to support the matching dynamics. This comparison shows that there is no or negligible change in dynamics upon H/D exchange. Coherent oscillations are pronounced at pH 7.5 and pD 7.8 due to a shorter system response of <40 fs. At pD 7.5 and pD 9.0 the system response was about 80 fs. Source data

An excited-state proton transfer from the chromophore to a water network of the protein should be affected by pH changes, altering the properties of chromophore–protein interactions. We thus compared the electronic dynamics following pH changes in the range from pH 6.6 to pH 8.4 (Extended Data Fig. 8). In Fig. 6a, transients at 12,345 cm^−1^ are presented for different pH values. We observed nearly identical bleaching contributions around time zero, assigned to stimulated emission (SE1) for all pH values. The GS* formation with 150 fs is more pronounced for increasing pH. The pH dependence of the signal amplitudes is plotted in the inset of Fig. 6a, showing a clear pH dependence with a p*K*_a_ value of 7.2 ± 0.3. Because the p*K*_a_ value of the chromophore is higher than 9 (Extended Data Fig. 8), we assign this pH dependence to the interaction with a surrounding amino acid. The p*K*_a_ value supports the interaction with a histidine, such as H248 or H278. Neither histidine is in direct contact with ring *C* or ring *D*, but instead they are in contact with water molecules and other amino acids. Because H248 is essential to stabilize the Pfr state, we investigated mutants at other positions, namely positions 278, 211 and 165. It has been shown previously that the impact of pH changes and altered amino acids on the excited-state dynamics reflects changes of the PES due to side-group interactions^19,52^. Given that we found here a correlation between pH and the properties of histidine that can be direct or indirect, we compare mutants possibly involved in the protein–water network to investigate its impact on the photoreaction dynamics. Upon H/D exchange we see no change in the ultrafast dynamics (Extended Data Fig. 8). Moreover, in the observed pH range the ELF absorption spectrum shows a redshift with increasing pH (Extended Data Fig. 8), supporting hydrogen bonding of the chromophore.

### Impact of site-specific mutants and pH/pD

**Extended Data Fig. 9:**
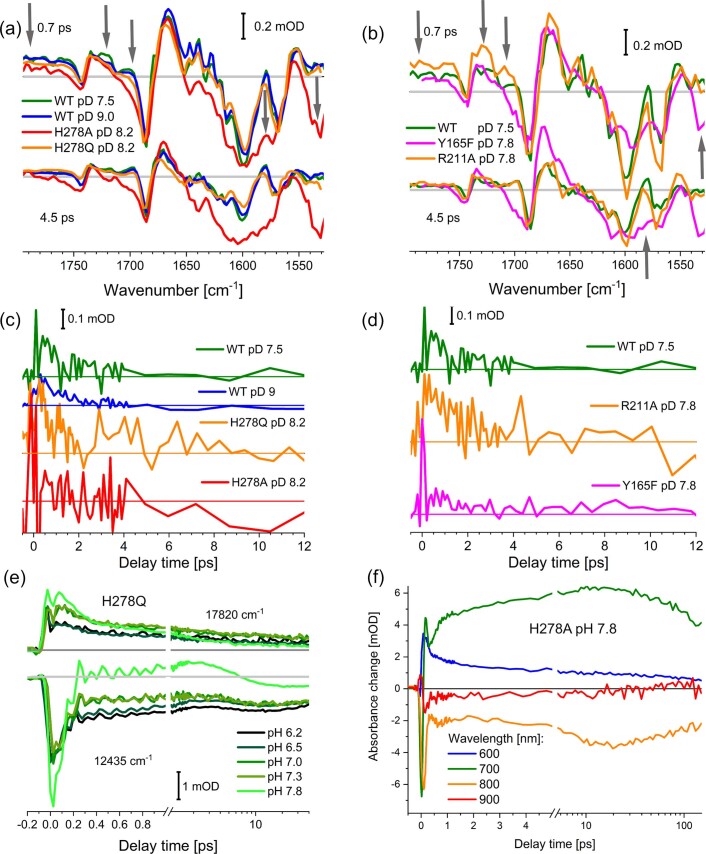
Agp2 variants. Agp2 variants: (**a-d**): Vis-pump IR-probe data; (a), and (b) scaled isotropic absorption difference spectra at averaged delay times around 0.7 ps (averaged from 0.4 to 1.0 ps) and 4.5 ps (averaged from 3.0 to 5.9 ps), normalized to the bleaching signal at 1689 cm^−1^ at 0.7 ps delay time: (a) the contribution of the CB at 1790 cm^−1^ is strongly reduced in Agp2-H278A at pD 8.2 in comparison to Agp2-H278Q at pD 8.2 and Agp2-WT. Moreover, the shoulder at 1727 cm^−1^ indicating a down-shift of prop*C* C = O stretching mode due to deprotonation of ring *C* or ring *D*, as well as the rising signal at 1695 cm^−1^ reflecting ELF formation are absent in Agp2-H278A (b) The absorbance difference spectra show nearly identical dynamics, except for the variants H278A at pD 8.2, and Y165F at pD 7.8. They deviate in the spectral shape around the CB, at the photoproduct marker band around 1700 cm^−1^, and in the C = C stretching region around 1580 cm^−1^, and 1530 cm^−1^, supporting a photoreaction with no or strongly reduced proton transfer upon altering the hydrogen-bonded network around Agp2-H278. ELF formation reflected by the positive marker band at 1695 cm^—1^ is not observed within hundreds of ps. (c) and (d) Vis-pump IR-probe transients averaged from 1800 to 1751 cm^−1^ of the CB taken from the dynamics presented in (a) and (b): (c) and (d) the decay of the CB signal in the first 2 ps is clearly visible except for H278A and Y165F at pD 7.8. A direct comparison of the dynamics of Y165F and H278A shows a substantially prolonged excited state lifetime. (e) Vis-pump Vis-probe transients at different pH values of Agp2-H278Q; isotropic transients of selected wavenumbers representing excited state absorption dynamics at 17820 cm^−1^; SE and GS* formation at 12435 cm^−1^. The measurements were performed one after another under the same experimental conditions. At low pH the contribution of sub-picosecond dynamics is very small. With pH the contribution of the fast sub-picosecond dynamics increases. The dynamics can be fitted with a sum of two models, the WT model (depicted in Fig. 3b) and an additional model with decay times on the tens to hundreds of picoseconds. (f) Vis-pump Vis-probe transients of H278A at pH 7.8; the excited state at 600 nm and the SE at 800 nm is still visible at 150 ps, reflecting dramatically prolonged excited state lifetimes. Source data

The vibrational difference spectra of Agp2-WT at pD 7.5 and 9.0, Agp2-H278Q at pD 8.2, Agp2-H278A at pD 8.2, Y165F at pD 7.8 and Agp2-R211A at pD 7.8 were investigated (Extended Data Fig. 9). The spectral shape and time-dependence of the dynamics are mainly identical, consistent with the far-reaching similarities of the respective ground-state resonance Raman (RR) and infrared (IR) difference spectra^28^. They only differ notably for Agp2-H278A at pD 8.2 and Agp2-Y165F at pD 7.8. Here, the contribution of the CB is strongly reduced, and the shoulder at 1,727 cm^−1^ indicating a downshift of the prop*C* C=O stretching mode due to deprotonation of ring *C* or ring *D*, as well as the rising signal at 1,695 cm^−1^ reflecting ELF formation, are missing. This can be justified by the missing hydrogen-bonding partner of H278 in Agp2-Y165F, and the inability of alanine to form hydrogen bonds in Agp2-H278A. Glutamine instead is able to participate in hydrogen bonds as a hydrogen-bond acceptor, but to a much lesser extent compared to histidine. The vibrational dynamics of Agp2-H278Q at pD 8.2 as well as the electronic dynamics at pH 7.8 (Fig. 6b) are similar to those in the Agp2-WT protein. On lowering the pH from 7.8 to 6.2, the photoreaction of Agp2-H278Q is substantially slowed down ~100 ps, similar to Agp2-H278A (Extended Data Fig. 9).

We assign the dynamics of the Agp2-H278Q mutant around pH 6.2 and the dynamics of Agp2-H278A around pD 8.2 to a photoreaction with no deprotonation in the electronic excited state (no bifurcation on the ES PES) and no conical intersection to the ground state leading to the ultrafast generation of the hot ground-state product GS*. Because we observe a substantial impact of Agp2-Y165F and Agp2-H278A variants on the photodynamics and CB contribution, we expect a water network in the close vicinity of H278 and Y165, and ring *C* or ring *D*, to act as a transient proton acceptor for the ultrafast photoreaction.

### Photoreaction of Pfr

**Extended Data Fig. 10:**
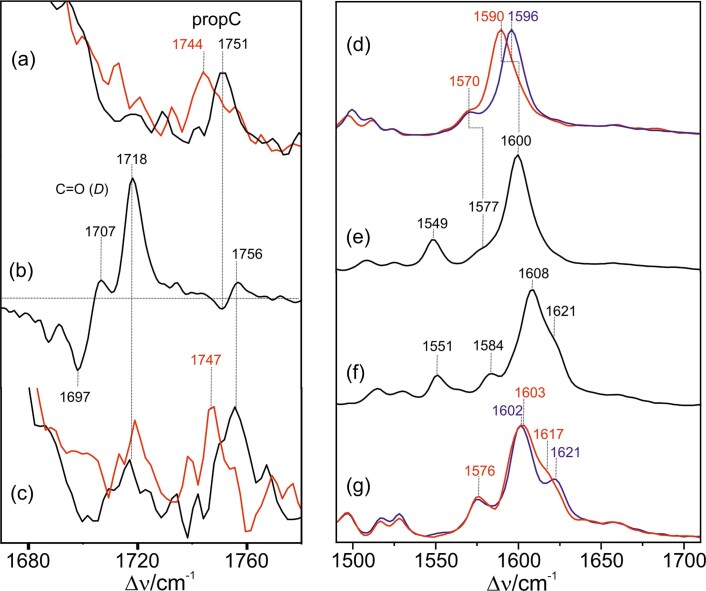
Vibrational spectra of Agp2-WT. Vibrational spectra of Agp2-WT. RR spectra of Agp2-WT, recorded at 90 K, in the Pfr (**a, d, e**) and Lumi-F state (**c, f, g**), cryogenically trapped at 140 K, together with the static FTIR difference spectrum “Lumi-F (positive bands) minus Pfr (negative bands)”, obtained at 90 K (b). Black and red lines represent the measurements in H_2_O and D_2_O, respectively. Blue lines refer to spectra obtained after H/D exchange in the dark, which is restricted to the rings *A*, *B*, and *C*^25^. In the IR difference spectrum (b), Lumi-F shows two positive peaks at 1718 and 1707 cm^−1^ in the ring *D* C = O stretching region. In Pfr, these modes are associated with a ca. 10-cm^—1^ H/D downshift such that the 1707 cm^−1^ signal may be related to that at 1695 cm^−1^ in the ultrafast experiments in D_2_O. The major 1718 cm^−1^ band, also identified in the RR spectrum of Lumi-F (c), shows no H/D sensitivity ruling out significant hydrogen bonding interactions and supporting its assignment to a C = O (*D*) conformation in Lumi-F at a later time that is not captured by the ultrafast IR experiment. This conclusion is consistent with the view that the cryogenically trapped photoproduct represents a late Lumi-F in contrast to the transient IR experiments probing the early Lumi-F. The RR spectra (d, e, f, g) mainly display modes governed by the methine bridge stretchings. The bands in this region also reflect the sequential H/D exchange that occurs instantaneously in the dark at rings *A*, *B*, and *C* but at ring *D* only after a photocycle^25^. This effect supports the vibrational assignments and allows concluding that in late Lumi−F (f, g) the photoisomerisation-induced structural changes comprise the entire chromophore. This conclusion is in agreement with a previous crystallographic study^80^. Source data

Next, we compared the ultrafast vibrational changes to the static vibrational spectra of the first intermediates that can be cryogenically trapped between 90 and 140 K (Extended Data Fig. 10). In the cryotrapped photoproducts, the prop*C* C=O stretching was detected at 1,747 cm^−1^ in D_2_O in the static RR spectra. It is distinctly higher in frequency than the signal in the transient IR spectrum at 1,735 cm^−1^ in Fig. 4. It is even higher in frequency than the corresponding mode of Pfr at 1,744 cm^−1^ (Extended Data Fig. 10). Altogether we thus conclude that the photoproducts identified in the transient IR experiments and the cryotrapping studies represent early and late Lumi-F states, respectively.

## Discussion

The present study demonstrates an ultrafast coherent photoreaction of Pfr in Agp2. The photoreaction is well described by at least two ultrafast components of 50 ± 50 fs and 150 ± 100 fs and two slower components of 1.5 ± 0.5 ps and 4.0 ± 0.8 ps. With a 50-fs time constant we see the formation of ~1,950 cm^−1^ redshifted stimulated emission (SE2), matching our calculated shifts of the SE for a deprotonated ring *D* of 2,089 cm^−1^ or ring *C* of 1,437 cm^−1^ in the ES. As shown in the scheme in Fig. 3b, an ultrafast relaxation and bifurcation on the excited-state PES occurs with 50 fs after excitation to S_1_, leading to fractions with protonated and deprotonated chromophores.

Strong coherent oscillations at frequencies around 300 and 340 cm^−1^ are visible in the dynamics. We tentatively assign the oscillations to the protonated ES that decays to the hot ground state GS* with damped oscillations. Thus, the 150-fs time constant reflects the population changes from the ES via conical intersections to GS* that relaxes back to Pfr with a decay time of 4 ps.

A fraction of ~50% is deprotonated in the ES within 50 fs. The proton or deuteron (in D_2_O) is transferred to a water network connected to H278, forming a vibrational CB within 300 fs. The fraction reacts forward to the protonated photoproduct ELF with an isomerized ring *D*. The reaction to ELF and GS* is accompanied with a reprotonation of the chromophore. Concomitant with ELF formation (overall yield of 35%) and ES decay, the CB decays. Because the CB is not observed in the Pfr ground state, we expect an altered electric field upon ES formation stabilizing the transient proton-loaded water network, and shaping the spectral properties of the CB (Fig. 5a). Our calculations demonstrate altered CB shapes in H_2_O and D_2_O networks on increasing the strength of the cylindrical confinement, which emulates the protein environment (Extended Data Fig. 6). With a specific confinement strength, both CBs in H_2_O and D_2_O are reproduced qualitatively (Fig. 5a). This suggests a water network causing the CB, in contrast to a shared proton between two contiguous groups.

We thus propose the following proton-coupled photoisomerization mechanism, also taking into account previous spectroscopic and structural data. In the electronic ground state of Pfr, ring *D*, ring *C* and prop*C* are protonated and prop*C* is hydrogen-bonded to Y165 and H278^6,25,27^. Upon photoexcitation, the electron density is increased at ring *B* and reduced at ring *C* and ring *D*, resulting in an electric field change. This field change alters the water network in the ES, as demonstrated in ultrafast X-ray studies^53–55^. The chromophore is deprotonated at ring *D* or at ring *C* and donates the proton to a water network connected with H278, stabilized by the electric field in the ES. We expect the proton acceptor site at ring *D* to rotate towards the transient water network in the ES. Subtle changes of the reaction coordinate, for example, by H278, result in a drastically altered photoreaction. Further computational studies have to be performed to address the detailed impact of these contributions to the photoreaction.

In the case of ring *C* deprotonation, the most probable proton transfer route is via W7 (Fig. 2) to H248δ, which becomes doubly protonated, shifting the SE to lower frequencies. Upon proton release from H248ε to the water network between prop*B* and prop*C* via W4, the CB is formed, and the SE should shift by 1,830 cm^−1^ to higher frequencies, according to our calculations. This blueshift is not observed (Fig. 3a). Concomitant with the decay of the ES, the proton is transferred back to ring *C* via the reverse route.

In the case of ring *D* deprotonation, two proton transfer routes seem to be plausible: one with and another without transient protonation of D196. If D196 is involved, the proton is transferred to the hydrogen-bonded D196 on the ultrafast timescale. The protonation of D196 is expected to redshift the SE at 12,900 cm^−1^ by 905 cm^−1^, matching the shoulder of the SE at 20-fs delay time (Fig. 3a). Upon photoexcitation, intermediate IR signals at 1,719 and 1,702 cm^−1^ are visible up to 0.5 ps (Fig. 4b) that could reflect protonation of D196, prior proton transfer to a water network between TW1 and TW2 (Fig. 5b) via the carbonyl group of ring *D* and formation of the CB. The transfer of the proton to the water network shifts the SE to ~10,900 cm^−1^ (SE2 in Fig. 3c). During the ES, ring *D* rotates clockwise, decreasing the distance between the nitrogen of ring *D* and the proton-loaded transient water network between TW1 and TW2. This enables reprotonation of ring *D* and crossing of the ES PES and ELF ground state. In the proton transfer route without D196 the proton is directly transferred from ring *D* via its carbonyl group to the transient water network between TW1 and TW2, reflected by the 50-fs rise of SE2 around 10,750 cm^−1^ and the rise of the CB within 300 fs (Figs. 3c and 4a). We favour the reaction pathway with deprotonation of ring *D* with or without involvement of D196. The impact of D196 has to be investigated in further studies^20^.

The dynamics alters upon pH changes with a p*K*_a_ of 7.2, which we assign to H278. The deprotonated H278 fosters the ultrafast generation of the hot ground state GS*, and induces changes in ES dynamics. We thus demonstrate the direct influence of the H278 side chain and its protonation status on the ES PES^19,52^. In Agp2-H278Q at low pH around 6.2 (Fig. 6b), formation of GS* and deprotonation of ring *D* or *C* is absent, and the excited-state lifetime is slowed 100-fold^28^.

These findings illustrate a possible way to increase the Pfr fluorescence QY by replacing the side chains participating in the protonated HBWN. Moreover, impeding the formation of GS* would increase the QY of the competing reaction pathways, such as fluorescence.

In a variety of phytochromes, the Pfr dynamics exhibit a bifurcation on the ES PES resulting in two lifetimes of ~1 ps and ~5 ps^14,19,56,57^. Here we demonstrate a bifurcation of the ES dynamics within 150 fs, resulting in two reaction channels, an ultrafast deactivation with 150 fs to a hot ground state subsequently cooling with ~4 ps, and an isomerization and reprotonation reaction with a time constant of 1.5 ps. The ES PESs governing the ES dynamics are sensitive to conformational changes around H278. Formation of ELF is linked to reprotonation of the chromophore, rotation of ring *D* and is accompanied by substantial changes of the hydrogen-bonding interactions of prop*C*, which seem to rule out a recovery of the initial prop*C*–Y165/H278 interactions. In fact, this interpretation is supported by the fact that in the variant Agp2-Y165F, the subsequent reaction cascade is arrested in the Meta-F state in which the protonated form of prop*C* is preserved^28^.

The ELF species must be considered as an early Lumi-F state, in comparison with the cryotrapped Lumi-F photoproducts, denoted as late Lumi-F. The transition from the early to the late Lumi-F states is associated with structural rearrangements in the chromophore binding pocket that transfer the C=O group of ring *D* and prop*C* into a different environment. Specifically, the upshift of the prop*C* carbonyl stretching in the late Lumi-F can be explained by strongly weakened hydrogen-bond interactions of the C=O group^58^. The pH dependence of the ELF spectrum, in contrast to Pfr, further supports ongoing interactions with the protein.

Furthermore, the present results strongly support the view of local electric-field changes as a key parameter for the protonation-linked structural changes in the Pfr → Pr phototransformation of Agp2. In fact, recent experimental and theoretical studies on Agp2 variants have demonstrated the impact on electric fields and hydrogen bonding for coupling chromophore and protein structural changes^27,28,59^. Here we have shown that the concerted interplay of ultrafast electric-field changes already in the ES connected to excited-state proton transfer to a protein–water network pave the way for the subsequent structural transformations in the ground state, thereby establishing a paradigm of functional protein–chromophore interactions.

## Methods

### Sample preparation

Agp2-WT, Agp2-H278Q, Agp2-H278A, Agp2-R211A and Agp2-Y165F mutants were expressed and assembled in vitro with BV as described previously^6,24,28,60^.

#### Molecular cloning of wild-type Agp2

The Agp2-PCM gene (NCBI Gen-Bank ID AAK87910) was PCR-amplified from *A. fabrum* genomic DNA and cloned into a pET21b expression vector with C-terminal His-tag using the following primers: forward primer sequence ATGTATATCTCCTTCTTAAAGTTAAAC and reverse primer sequence CATCACCATCACCATCACTAAGATCCG. The gene encoding the photosensor core module (PCM) derived from Agp2 (1–501 amino acids plus hexa-histidine tag) was transformed into *Escherichia coli* BL21-DE3 cells (Agilent Technologies)^6,23^.

#### Protein expression and purification of wild-type Agp2-PCM

The Agp2-PCM construct was expressed using an auto-induction medium (Overnight Express Instant TB Medium, Novagen) for 48 h and 20 °C. Cell pellets were washed and cell lysis was carried out using cell fluidizer (Microfluidics) in 50 mM Tris-HCl buffer containing 50 mM NaCl at pH 7.8, 5% glycerol, 2 mg ml^−1^ lysozyme (Merck Millipore), 60 μg ml^−1^ DNAse (Sigma-Aldrich), 1 mM MgCl_2_ and 0.5 mM phenylmethanesulfonyl fluoride (Sigma-Aldrich). Lysed cells were centrifuged and protein in the supernatant was precipitated with 2 M ammonium sulfate. The pellet was dissolved with 50 mM Tris/HCl, 10 mM imidazole, 400 mM NaCl at pH 7.8 and loaded with the same buffer onto a Ni-NTA column (5-ml HP columns; GE Healthcare). Purified apo-protein was eluted with a linear imidazole gradient. Imidazole was removed by ammonium sulfate precipitation. The chromophore BV (Frontier Scientific) was added at ~3× molar excess to the apo-protein. The final holo-protein was concentrated by ammonium sulfate precipitation and rediluted in size-exclusion buffer (20 mM HEPES buffer, pH 7.5, 150 mM NaCl). Size-exclusion chromatography (HiLoad Superdex 200 column, GE Healthcare) was performed and yielded pure holo-protein at a concentration of 30 mg ml^−1^. The buffer of the pure holo-protein solutions was changed using Amicon ultra centrifugal filters (30 kDa, Merck Millipore). To remove the buffer, the Agp2-PCM sample was concentrated. The protein samples were then washed with D_2_O buffer (containing 20 mM HEPES, 150 mM NaCl, pD 7.8). For measurements of the protein samples in D_2_O buffer, the samples were concentrated to a concentration of 62–145 mg ml^−1^, then the protein absorption at a wavelength of 750 nm was measured. This resulted in an absorption of 0.4–1.4 OD in samples with 0.1-mm thickness. All samples were measured directly after concentration. The Pfr–Pr–Pfr photocycles test and visible absorption spectra of the samples before and after the experiments show that the samples were stable during the pump–probe measurements.

### Computational methods

The three-dimensional structural models of the Agp2 in Pfr and Lumi-F states were generated using the crystal structure with PDB 6G1Y as template. The SWISS MODEL server^61^ was used to reconstruct the gaps V80-T85 and G120-A123 via a homology modelling technique, and the Karlsberg2+ server^62^ was used to insert hydrogen atoms into the initial structure and for assigning the protonation and tautomeric state of titratable residues based on electrostatic calculations. In particular, H248 and H278 were initially modelled as charge-neutral with a proton on the Nε^7^. The BV chromophore was modelled with protons on all pyrrole rings and a protonated propionic side chain *C* (prop*C*) to account for previous and current spectroscopic data^27^. After energy minimization and thermal equilibration of the solvated protein systems, the geometry of the chromophore binding sites was optimized using a hybrid QM/MM approach combining density functional theory and the CHARMM32b2 force field. The active region in the optimization process included all atoms within a 20-Å radius of N22 of the BV cofactor. Among them, the entire BV moiety, the side chains of Cys13 and the pyrrole water were treated at the B3LYP/6-31G* level.

The second-order approximate coupled-cluster model CC2^63^ with resolution-of-identity approximation (RI) was used for calculation of excitation energies and dipole moments for the five lowest excited states. As the basis set we used cc-pVDZ with the corresponding auxiliary basis set^64^. Input files for the computations were generated with the Amber QM/MM interface^65–67^. The excitation energies and associated TDMs (13) were computed with Turbomole (version 7.3)^68,69^. Particle–hole distances and transition densities were obtained from the RI-CC2 results using TheoDORE (version 1.7.3) (http://theodore-qc.sourceforge.net/) with ORBKIT^70^.

Excitation energies for the S_0_ → S_1_ transitions were computed using RI-CC2/cc-pVDZ for different models with protonated and deprotonated pyrrole rings by transferring the proton to the bulk (that is, removing it) or to adjacent amino-acid side chains (Extended Data Fig. 1).

### Femtosecond experiments

Agp2 samples at different pD and pH values were placed between two CaF_2_ windows in a sample holder with a 50-, 100- or 200-µm Teflon spacer. All experiments were performed at room temperature. The optical density at 750 nm was between 0.2 and 1. The sample was moved continuously by a Lissajous sample holder to ensure a fresh sample volume between two pump pulses. We used background illumination around 680 nm (laser diode). Mostly, a small number of delay times were measured before time zero, not enough to perform exhaustive investigations of the perturbed free induction decay (PFID). We detected several data points around −60 ps to measure the background signal without the influence of the PFID. Data acquisitions were performed in series of delay times with a fixed number of shots (typically 1,000) at each delay time. Each series was saved individually as a scan. We used a median filter to exclude outliers (10% were cut off). We used a repetition rate of 1 kHz and a chopper to block every second pump pulse.

VIS-pump VIS-supercontinuum-probe experiments were performed using a home-built single-stage non-collinear optical parametric amplifier pumped at 515 nm (Pharos laser system, 1,030 nm). The tunable pump pulse was between 740 and 780 nm, and the supercontinuum was generated by focusing the fundamental at 1,030 nm into a sapphire window. The pump pulse was modulated by a Dazzler (Fastlite) to achieve high time resolution. In the experiments, the system response was between 40 and 80 fs (full-width at half-maximum (FWHM)). The pump energy was around 200 to 300 nJ with pump focus of ~250 µm (10–90%). Broadband detection in the visible was performed using a Shamrock 303i spectrograph and a 2,048-pixel charge-coupled device (CCD; resolution below 1 nm)^71^.

The VIS-pump IR-probe experiments were performed in a home-built set-up^72^. We excited the sample with a pump pulse at 765 nm, ~110 fs, 400–600 nJ energy, and a focus of 300 µm. The system response was between 200 and 300 fs. We used excitation efficiencies below 15 to 20%, enabling photoselection experiments. We used two mid-IR probe beams to probe the same sample spot 1.5 ns before and femtoseconds to picoseconds after VIS excitation^72,73^. Probe pulses were dispersed by an imaging spectrograph and recorded with either a 2 × 32 element mercury cadmium telluride (MCT) array detector (1.5-cm^−1^ resolution) or a 128 × 128 MCT CCD (~2.5-cm^−1^ resolution). This referencing increases the signal-to-noise ratio by a factor of three. The system response was ~250 fs in the spectral region from 1,900 to 1,525 cm^−1^. Here, we cannot resolve possible coherent oscillations due to the limited time resolution. The vibrational data exhibit a lower signal-to-noise ratio compared to the visible data. We found global decay constants between 1 and 2 ps and between 3.5 and 5 ps. Thus, we successfully simulated the vibrational data with the same global decay constants of 150 fs, 1.5 ps and 4 ps used for the electronic dynamics (Extended Data Fig. 5).

Polarization-resolved femtosecond VIS-pump broadband VIS probe and VIS-pump IR-probe experiments were performed by changing the pump pulse polarization with respect to the probe pulse polarization between every scan. Isotropic data (iso) were generated by iso = (par+2*per)/3. The complete Pfr Agp2-WT dataset is simulated by a global fit with time constants of 50 ± 50 fs, 150 ± 100 fs, 1.5 ± 0.5 ps and 4.0 ± 0.8 ps (Extended Data Figs. 3 and 4). The data were mainly analysed with Python programs using the package skultrafast^74^.

### Ab initio Born–Oppenheimer MD simulations

Ab initio Born–Oppenheimer MD simulations were performed with CP2K 6.1 using a polarizable double-zeta basis set, optimized for small molecules, for the valence electrons (DZVP-MOLOPT-SR-GTH), dual-space pseudopotentials, the BLYP exchange-correlation functional and D3 dispersion correction^75–77^. The cutoff for the plane-wave representation was optimized to 400 Ry. For simulations in the NVT (fixed particle number N, fixed Volume V, and fixed temperature T) ensemble, temperature was controlled at 300 K by coupling all atoms to a stochastic velocity rescaling thermostat with a 10-fs time constant^78^.

The spectral power densities *ωχ*″(*ω*), where *χ*″(*ω*) is the imaginary part of the Fourier-transformed dielectric susceptibility and *ω* = 2π*f*, are calculated from the autocorrelation of the total dipole moment *p*, obtained from atom positions and Wannier centres that are localized at each eighth simulation step. Linear response theory relates the dielectric susceptibility *χ*(*t*) to the equilibrium autocorrelation of the dipole moment $C(t) = {\langle {p(t)p(0)}\rangle}$, reading in Fourier space

$${\chi^{\prime\prime} \left( \omega \right)} = {\frac{\omega }{{2{k}_{{{\mathrm{B}}}}{TV}\epsilon _0}}}{\mathop {\sum }\limits_{{{\mathrm{l}}}}} {C}{(\omega )}$$

where *ε*_0_ is the vacuum permittivity and *V* is the effective system volume.

The first simulated system models two water molecules confined between the carboxylic side chains of prop*B* and prop*C* and consists of two deprotonated carboxylic acid molecules, two water molecules and an excess proton, as shown in Extended Data Fig. 6. The total charge amounts to −1*e*. The carbon atoms were constrained at a fixed mutual distance of 7.4 Å taken from the crystal structure^6^. Simulations were performed both for H_2_O and D_2_O by replacing all hydrogen atoms in the systems by deuterium atoms. The simulation box size is 22 × 12 × 12 Å^3^ and an effective volume of 0.3 nm^3^ was assumed for calculation of the spectra. The molecules were kept near the central axis of the simulation box by fully constraining the carbon atoms. The water oxygen atoms were constraint by a quadratic potential in the *y* and *z* direction, *k*(*y*^2^ + *z*^2^), illustrated in Extended Data Fig. 6, to keep them close to the central axis and to model the confining effect inside the protein. Because the realistic confinement strength was not known, simulations at three different strengths were performed at *k* = 0.0, 0.4, 4.0 *k*_B_*T*/Å^2^, where *k*_B_*T* is the thermal energy. For each strength and water isotope a 20-ps simulation was performed under NVT conditions at 300 K using a simulation time step of 0.5 fs. Consequently, five independent 20-ps simulations were performed under NVE (fixed particle number N, fixed volume V, fixed energy E) conditions using a time step of 0.25 fs and starting from different random snapshots of the NVT data.

The second model system consists of one side of the chromophore including ring *C* and ring *D*, side chains H278 and Y165, which were each truncated at the ring, and three water molecules TW1, TW2 (Fig. 5b) and W6 (Fig. 2). An illustration is given in Extended Data Fig. 6. The initial nuclear coordinates are taken from the classical MD simulations starting from the crystal structure with an enolic ring *D* (Fig. 5b). The model describes the scenario of a deprotonated ring *D* and was therefore set up with a neutral deprotonated ring *D*, but also an excess proton and thus a total charge of +1*e*. The excess proton was initially placed between water molecule TW2 and the carboxylic side chain of ring *C*, which was found by initial equilibration to be a metastable configuration. Again, simulations for H_2_O and D_2_O were performed. In the case of D_2_O, the water hydrogen atoms and the exchangeable hydrogen atoms at the protein side chains were replaced by deuterium atoms. The simulation box size was 20 × 20 × 20 Å^3^ and an effective volume of 0.8 nm^3^ was assumed for calculation of the spectra. A number of constraints were applied to model the confining effect inside the protein. The heavy atoms of the chromophore and H278 were fully constrained, except for the carboxylic side chain at ring *C*. The water oxygen atoms were each constrained by a weak quadratic potential in all dimensions, *k*(*x*^2^ + *y*^2^ + *z*^2^), with *k* = 0.04 *k*_B_*T*/Å^2^. Furthermore, for Y165, the oxygen atom of the hydroxyl group and the carbon atom of the phenyl ring that connects to the backbone of the protein were constrained by a quadratic potential with *k* = 0.04 *k*_B_*T*/Å^2^. A small number of simulations of this non-equilibrium system were performed for H_2_O and D_2_O under NVT conditions at 300 K using a simulation time step of 0.5 fs. All showed a transfer of the excess proton to Y165 as a Grotthuss process within the first 5 ps; in some simulations the excess proton was further transferred to the nitrogen atom of the keto group at ring *D*. This is illustrated in Extended Data Fig. 6. Interestingly, in some simulations, Y165 was protonated at the phenyl ring, before the proton from the hydroxyl group was released, which probably presents another metastable state. Each simulation was run for ~5 ps to 7.5 ps or until the keto group at ring *D* was protonated, which was considered the metastable reference configuration. The first 0.1 ps were truncated for initial fast equilibration.

### Static vibrational spectroscopy

For the Fourier transform infrared spectroscopy (FTIR) measurements, the protein samples were placed between two BaF_2_ windows in a sample holder. The samples were cooled to 130 K with an OptistatTN cryostat, and FTIR spectra were recorded by an MCT detector. Three 785-nm laser diodes were used to induce the Pfr to Lumi-F conversion. The recording time was 2 min before (Pfr) and 2 min during illumination (Lumi-F) with a spectral resolution of 2 cm^−1^. Difference spectra were calculated by subtracting the Pfr spectrum from the Lumi-F spectrum. Cryogenic RR spectroscopy was carried out as described previously. Further experimental details are given elsewhere^28^.

## Online content

Any methods, additional references, Nature Research reporting summaries, source data, extended data, supplementary information, acknowledgements, peer review information; details of author contributions and competing interests; and statements of data and code availability are available at 10.1038/s41557-022-00944-x.

## Data Availability

The raw data for the VIS-pump VIS-probe and VIS-pump IR-probe results of Figs. 3, 4 and 6 are available at https://box.fu-berlin.de/s/3XBnLjqqeWRC9Nn. Source data are provided with this paper.

## Source data

Source Data Fig. 3 Data for Fig. 3a and 3c (ascii format).
Source Data Fig. 4 Data for Fig. 4a and 4b and insets (ascii format).
Source Data Fig. 5 Data for Fig. 5a (ascii format).
Source Data Fig. 6 Data for Fig. 6a and 6b and inset (ascii format).
Source Data Extended Data Fig. 2 Data for Extended Data Fig. 2a, 2c, and 2d (ascii format).
Source Data Extended Data Fig. 3 Data for Extended Data Fig. 3a, 3b, and 3c (ascii format).
Source Data Extended Data Fig. 4 Data for Extended Data Fig. 4a, 4b, and 4c (ascii format).
Source Data Extended Data Fig. 5 Data for Extended Data Fig. 5a, 5b, and 5c (ascii format).
Source Data Extended Data Fig. 6 Data for the two calculated networks in H_2_O and D_2_O (ascii format and csv format).
Source Data Extended Data Fig. 8 Data for Extended Data Fig. 8a, 8b, 8c, and 8d (ascii format).
Source Data Extended Data Fig. 9 Data for Extended Data Fig. 9a, 9b, 9c, 9d, 9e, and 9f (ascii format).
Source Data Extended Data Fig. 10 Data for Extended Data Fig. 10 a-g (ascii format).
